# Supplementary material for: Variable selection using a smooth information criterion for distributional regression models
Source: Stat Comput. 2023 Apr 21;33(3):71. doi: 10.1007/s11222-023-10204-8 (PMC10121547; doi:10.1007/s11222-023-10204-8)
Supplement: Supplementary file 1 — (pdf 212 KB) [file 11222_2023_10204_MOESM1_ESM.pdf]

Supplementary Material for "Variable Selection Using a Smooth Information Criterion for Distributional Regression Models"

Meadhbh O'Neill[1*] and Kevin Burke[1]

[1*]Department of Mathematics and Statistics, University of Limerick, Republic of Ireland.

*Corresponding author(s). E-mail(s): meadhbh.oneill@ul.ie;
Contributing authors: kevin.burke@ul.ie;

Appendix A Simulation Results: Fixed Smoothing Parameter ϵ

This section contains additional simulation results for the MPR-SIC method. The smoothing parameter ϵ is fixed at a single value (i.e., the ϵ-telescoping procedure is not performed). The smoothing parameter is fixed at $\epsilon = 10^{-1}$, 10^{-2}, and 10^{-3}.

- Table A1 is analogous to Table 4 of the main paper, but showing the model selection metrics when the smoothing parameter ϵ is fixed at a single value. The variable selection performance is poor for all values of ϵ that are tested.
- Table A2 in analogous to Table 5 of the main paper, but showing the estimation and inference metrics when the smoothing parameter ϵ is fixed at a single value. The estimation and inferential performance for the location component of the model does not appear to be impacted. However, for $\epsilon = 10^{-2}$ and 10^{-3}, the estimation and inferential performance for the dispersion component does not perform well — the estimated values do not move from the initial values that are used in the optimization.

Table A1: Simulation results: model selection metrics for MPR-SIC with fixed smoothing parameter ϵ

		$\epsilon = 10^{-1}$				$\epsilon = 10^{-2}$				$\epsilon = 10^{-3}$			
	n	C(6)	IC(0)	PT	MSE	C(6)	IC(0)	PT	MSE	C(6)	IC(0)	PT	MSE
β	100	0.00	0.00	0.00	1.59	0.00	0.00	0.00	2.48	0.00	0.00	0.00	2.52
	500	0.00	0.00	0.00	0.12	0.00	0.00	0.00	0.36	0.01	0.00	0.00	0.53
	1000	0.00	0.00	0.00	0.05	0.00	0.00	0.00	0.13	0.01	0.00	0.00	0.27
α	100	0.31	0.31	0.00	3.42	0.47	0.47	0.00	6.21	0.08	0.07	0.00	6.21
	500	0.01	0.01	0.00	0.35	0.13	0.13	0.00	4.49	0.33	0.32	0.00	7.01
	1000	0.03	0.03	0.00	0.16	0.02	0.02	0.00	1.41	0.46	0.45	0.00	7.09

C, average correct zeros; IC, average incorrect zeros; PT, the probability of choosing the true model; MSE, the average mean squared error.

Table A2: Simulation results: estimation and inference metrics for MPR-SIC with fixed smoothing parameter ϵ

$\epsilon = 10^{-1}$		$n = 100$				$n = 500$				$n = 1000$			
	θ	$\hat{\theta}$	SE	SEE	CP	$\hat{\theta}$	SE	SEE	CP	$\hat{\theta}$	SE	SEE	CP
β_0	0.0	0.04	0.67	1.94	0.93	0.01	0.17	0.30	0.92	-0.00	0.12	0.08	0.93
β_1	1.0	1.00	0.53	0.87	0.87	0.99	0.10	0.13	0.93	1.00	0.07	0.04	0.94
β_2	0.5	0.52	0.64	1.27	0.72	0.50	0.19	0.17	0.90	0.50	0.10	0.06	0.93
β_3	0.5	0.46	0.36	1.10	0.82	0.50	0.08	0.09	0.94	0.50	0.03	0.03	0.94
β_4	1.0	0.99	0.41	0.68	0.90	1.00	0.06	0.08	0.93	1.00	0.03	0.03	0.93
β_5	0.5	0.46	0.36	0.66	0.81	0.50	0.08	0.08	0.93	0.50	0.04	0.03	0.94
β_6	1.0	0.95	0.77	1.43	0.75	0.98	0.27	0.54	0.91	1.00	0.14	0.09	0.90
α_0	0.0	1.43	0.62	0.91	0.28	0.14	0.44	0.41	0.82	0.06	0.34	0.13	0.83
α_1	0.5	0.12	0.22	0.27	0.35	0.45	0.12	0.11	0.85	0.48	0.08	0.06	0.89
α_2	1.0	0.16	0.30	0.41	0.26	0.88	0.32	0.43	0.83	0.95	0.20	0.28	0.90
α_3	0.5	0.06	0.16	0.24	0.17	0.44	0.17	0.34	0.88	0.48	0.09	0.07	0.89
α_4	1.0	0.24	0.34	0.41	0.33	0.91	0.21	0.15	0.79	0.96	0.14	0.06	0.85
α_7	0.5	0.09	0.20	0.28	0.27	0.45	0.13	0.16	0.89	0.48	0.08	0.06	0.89
α_8	1.0	0.24	0.35	0.49	0.30	0.91	0.22	0.24	0.77	0.96	0.15	0.06	0.84
$\epsilon = 10^{-2}$		$n = 100$				$n = 500$				$n = 1000$			
	θ	$\hat{\theta}$	SE	SEE	CP	$\hat{\theta}$	SE	SEE	CP	$\hat{\theta}$	SE	SEE	CP
β_0	0.0	-0.01	0.78	2.04	0.94	-0.00	0.31	0.67	0.91	0.01	0.19	0.28	0.90
β_1	1.0	1.05	0.69	0.52	0.79	1.00	0.24	0.28	0.78	0.99	0.11	0.09	0.90
β_2	0.5	0.54	0.76	1.04	0.78	0.49	0.29	0.36	0.87	0.50	0.13	0.14	0.93
β_3	0.5	0.49	0.37	1.21	0.87	0.49	0.14	0.19	0.97	0.50	0.06	0.08	0.98
β_4	1.0	1.01	0.53	0.42	0.84	1.00	0.18	0.22	0.86	1.00	0.08	0.07	0.92
β_5	0.5	0.49	0.41	0.47	0.82	0.49	0.14	0.26	0.94	0.50	0.06	0.06	0.95
β_6	1.0	0.97	0.82	1.25	0.83	1.00	0.32	1.61	0.91	1.00	0.19	0.20	0.91
α_0	0.0	2.25	0.60	0.15	0.00	1.81	0.74	0.30	0.07	0.70	0.82	0.27	0.34
α_1	0.5	0.00	0.00	0.00	0.00	0.11	0.20	0.05	0.17	0.36	0.21	0.07	0.67
α_2	1.0	0.00	0.00	0.00	0.00	0.24	0.41	0.08	0.20	0.75	0.41	0.20	0.69
α_3	0.5	0.00	0.00	0.00	0.00	0.03	0.09	0.01	0.03	0.14	0.22	0.04	0.25
α_4	1.0	0.00	0.00	0.00	0.00	0.25	0.42	0.05	0.21	0.77	0.39	0.09	0.68
α_7	0.5	0.00	0.00	0.00	0.00	0.06	0.16	0.02	0.09	0.29	0.24	0.08	0.56
α_8	1.0	0.00	0.00	0.00	0.00	0.24	0.41	0.11	0.19	0.77	0.39	0.09	0.68
$\epsilon = 10^{-3}$		$n = 100$				$n = 500$				$n = 1000$			
	θ	$\hat{\theta}$	SE	SEE	CP	$\hat{\theta}$	SE	SEE	CP	$\hat{\theta}$	SE	SEE	CP
β_0	0.0	-0.01	0.78	0.77	0.93	0.02	0.38	0.55	0.92	0.00	0.27	0.27	0.90
β_1	1.0	1.05	0.69	0.37	0.78	1.00	0.34	0.19	0.73	1.00	0.25	0.12	0.73
β_2	0.5	0.54	0.76	0.60	0.85	0.50	0.33	0.33	0.89	0.50	0.23	0.20	0.90
β_3	0.5	0.50	0.37	0.43	0.94	0.50	0.16	0.21	0.98	0.50	0.11	0.14	0.99
β_4	1.0	1.01	0.54	0.35	0.83	1.00	0.24	0.18	0.84	1.00	0.17	0.12	0.84
β_5	0.5	0.49	0.42	0.35	0.87	0.49	0.17	0.18	0.94	0.50	0.12	0.12	0.95
β_6	1.0	0.97	0.82	0.75	0.89	1.00	0.35	0.46	0.96	0.99	0.25	0.28	0.95
α_0	0.0	2.25	0.60	0.14	0.00	2.54	0.34	0.06	0.00	2.57	0.27	0.04	0.00
α_1	0.5	0.00	0.00	0.00	0.00	0.00	0.02	0.00	0.00	0.00	0.02	0.00	0.00
α_2	1.0	0.00	0.00	0.00	0.00	0.00	0.00	0.00	0.00	0.00	0.03	0.00	0.00
α_3	0.5	0.00	0.00	0.00	0.00	0.00	0.00	0.00	0.00	0.00	0.02	0.00	0.00
α_4	1.0	0.00	0.00	0.00	0.00	0.00	0.00	0.00	0.00	0.00	0.03	0.00	0.00
α_7	0.5	0.00	0.00	0.00	0.00	0.00	0.00	0.00	0.00	0.00	0.02	0.00	0.00
α_8	1.0	0.00	0.00	0.00	0.00	0.00	0.00	0.00	0.00	0.00	0.00	0.00	0.00

SE, standard deviation of estimates over 1000 replications; SEE, average of estimated standard errors over 1000 replications; CP, the empirical coverage probability of a nominal 95% confidence interval.

Appendix B Simulation Results: Fewer steps T in the ϵ-telescope

This section contains additional simulation results for the MPR-SIC method, where fewer steps T are implemented in the ϵ-telescope. Results for sequences of $T = 50$ and $T = 10$ steps.

- Table B3 is analogous to Table 4 of the main paper, but showing the model selection metrics for fewer steps T in the ϵ-telescope. For $T = 50$, the performance is comparable with the performance when $T = 100$. When $T = 10$, the model selection metrics are poor as the zero coefficients are not being driven close enough to zero.
- Table B4 is analogous to Table 5 of the main paper, but gives the estimation and inference metrics when fewer steps T are used in the ϵ-telescope. Estimation and inferential results are comparable with the case when $T = 100$.

Table B3: Simulation results: model selection metrics for MPR-SIC with fewer steps T

		$T = 50$				$T = 10$			
	n	C(6)	IC(0)	PT	MSE	C(6)	IC(0)	PT	MSE
β	100	5.14	0.14	0.43	0.14	0.49	0.00	0.00	0.20
	500	5.86	0.00	0.88	0.01	0.32	0.00	0.00	0.02
	1000	5.92	0.00	0.94	0.00	0.34	0.00	0.01	0.01
α	100	5.42	0.71	0.32	0.63	0.43	0.02	0.00	0.97
	500	5.89	0.00	0.91	0.04	0.24	0.00	0.00	0.06
	1000	5.92	0.00	0.94	0.02	0.27	0.00	0.00	0.03

C, average correct zeros; IC, average incorrect zeros; PT, the probability of choosing the true model; MSE, the average mean squared error.

Table B4: Simulation results: estimation and inference metrics for MPR-SIC with fewer steps T

$T = 50$

	θ	$n = 100$				$n = 500$				$n = 1000$			
		$\hat{\theta}$	SE	SEE	CP	$\hat{\theta}$	SE	SEE	CP	$\hat{\theta}$	SE	SEE	CP
β_0	0.0	0.01	0.22	0.13	0.77	-0.00	0.06	0.05	0.93	-0.00	0.04	0.04	0.94
β_1	1.0	1.00	0.15	0.10	0.77	1.00	0.04	0.04	0.93	1.00	0.03	0.03	0.96
β_2	0.5	0.48	0.23	0.10	0.72	0.50	0.05	0.05	0.92	0.50	0.03	0.03	0.94
β_3	0.5	0.49	0.11	0.07	0.79	0.50	0.03	0.03	0.94	0.50	0.02	0.02	0.94
β_4	1.0	1.00	0.11	0.07	0.80	1.00	0.03	0.03	0.94	1.00	0.02	0.02	0.93
β_5	0.5	0.50	0.11	0.07	0.77	0.50	0.03	0.03	0.93	0.50	0.02	0.02	0.95
β_6	1.0	1.01	0.24	0.11	0.71	1.00	0.05	0.05	0.91	1.00	0.04	0.03	0.94
α_0	0.0	-0.18	0.44	0.23	0.66	-0.03	0.11	0.10	0.90	-0.02	0.07	0.07	0.91
α_1	0.5	0.45	0.28	0.13	0.73	0.50	0.07	0.07	0.93	0.50	0.05	0.05	0.94
α_2	1.0	1.07	0.37	0.17	0.77	1.01	0.08	0.07	0.92	1.01	0.05	0.05	0.93
α_3	0.5	0.52	0.36	0.14	0.59	0.51	0.08	0.08	0.95	0.51	0.05	0.05	0.94
α_4	1.0	1.11	0.24	0.17	0.82	1.01	0.07	0.07	0.95	1.01	0.05	0.05	0.94
α_7	0.5	0.51	0.28	0.14	0.73	0.51	0.06	0.07	0.96	0.50	0.05	0.05	0.94
α_8	1.0	1.11	0.24	0.17	0.81	1.01	0.07	0.07	0.93	1.01	0.05	0.05	0.93

$T = 10$

	θ	$n = 100$				$n = 500$				$n = 1000$			
		$\hat{\theta}$	SE	SEE	CP	$\hat{\theta}$	SE	SEE	CP	$\hat{\theta}$	SE	SEE	CP
β_0	0.0	0.01	0.26	0.18	0.79	-0.00	0.08	0.08	0.93	-0.00	0.05	0.05	0.94
β_1	1.0	1.00	0.17	0.11	0.78	1.00	0.05	0.05	0.93	1.00	0.03	0.03	0.95
β_2	0.5	0.50	0.20	0.13	0.79	0.50	0.06	0.05	0.92	0.50	0.03	0.03	0.95
β_3	0.5	0.49	0.12	0.08	0.80	0.50	0.03	0.03	0.93	0.50	0.02	0.02	0.94
β_4	1.0	1.00	0.13	0.08	0.81	1.00	0.03	0.03	0.95	1.00	0.02	0.02	0.93
β_5	0.5	0.51	0.11	0.08	0.78	0.50	0.03	0.03	0.93	0.50	0.02	0.02	0.94
β_6	1.0	1.00	0.25	0.16	0.79	1.00	0.07	0.07	0.93	1.00	0.05	0.04	0.93
α_0	0.0	-0.43	0.48	0.35	0.69	-0.06	0.13	0.13	0.89	-0.03	0.09	0.10	0.92
α_1	0.5	0.54	0.25	0.19	0.88	0.50	0.07	0.07	0.93	0.50	0.05	0.05	0.95
α_2	1.0	1.17	0.44	0.32	0.83	1.03	0.11	0.12	0.93	1.01	0.08	0.08	0.94
α_3	0.5	0.64	0.32	0.22	0.78	0.51	0.08	0.08	0.94	0.51	0.05	0.05	0.94
α_4	1.0	1.17	0.27	0.20	0.79	1.02	0.07	0.07	0.94	1.01	0.05	0.05	0.94
α_7	0.5	0.59	0.26	0.20	0.85	0.51	0.07	0.07	0.96	0.50	0.05	0.05	0.94
α_8	1.0	1.18	0.27	0.20	0.79	1.02	0.07	0.07	0.92	1.01	0.05	0.05	0.94

SE, standard deviation of estimates over 1000 replications; SEE, average of estimated standard errors over 1000 replications; CP, the empirical coverage probability of a nominal 95% confidence interval.

Appendix C Simulation Results: Single Parameter Setting

This section contains the results of a simulation study carried out in a single parameter setting. Note that fewer replicates for the BAMLSS procedure are used (200 replicates) due to the computational intensity of the method. The other approaches are averaged over 1000 replicates.

- Table C5 is analogous to Table 2 of the main paper, but showing the true values that are used to simulate homoscedastic data.
- Table C6 is analogous to Table 6 of the main paper, but showing the out-of-sample prediction coverage probabilities from the single parameter setting.
- Table C7 is analogous to Table 4 of the main paper, but showing the model selection metrics from the single parameter setting.
- Table C8 is analogous to Table 5 of the main paper, but showing the estimation and inference metrics from the single parameter setting.

Table C5: True parameter values

		E	M	B	N	N	M	N	N	M	B	E	M
	X_0	X_1	X_2	X_3	X_4	X_5	X_6	X_7	X_8	X_9	X_{10}	X_{11}	X_{12}
β	0	1	0.5	0.5	1	0.5	1	0	0	0	0	0	0
α	0	0	0	0	0	0	0	0	0	0	0	0	0

E = Exponential, B = Bernoulli, N = independent normal,
M = multivariate normal (correlated).

Table C6: Out-of-sample prediction coverage probabilities

	MPR-SIC			BAMLSS			SPR-SIC			ALASSO-IC		
n	100	500	1000	100	500	1000	100	500	1000	100	500	1000
Overall	0.90	0.94	0.95	0.89	0.94	0.95	0.92	0.95	0.95	0.93	0.95	0.95

Out-of-sample coverage is calculated for a sample 20% the size of the original data.

Table C7: Model selection metrics

		MPR-SIC				BAMLSS			
	n	C(6)	IC(0)	PT	MSE	C(6)	IC(0)	PT	MSE
β	100	5.64	0.27	0.55	0.12	5.41	0.34	0.40	0.17
	500	5.93	0.00	0.94	0.02	5.63	0.00	0.68	0.03
	1000	5.95	0.00	0.95	0.01	5.64	0.00	0.71	0.01
	n	C(12)	IC(0)	PT	MSE	C(12)	IC(0)	PT	MSE
α	100	11.26	0.00	0.51	0.24	11.01	0.00	0.46	0.49
	500	11.87	0.00	0.88	0.01	11.28	0.00	0.52	0.06
	1000	11.91	0.00	0.92	0.00	11.39	0.00	0.62	0.03
		SPR-SIC				ALASSO-IC			
	n	C(6)	IC(0)	PT	MSE	C(6)	IC(0)	PT	MSE
β	100	5.75	0.25	0.60	0.10	5.57	0.18	0.55	0.11
	500	5.94	0.00	0.94	0.02	5.91	0.00	0.92	0.02
	1000	5.95	0.00	0.95	0.01	5.95	0.00	0.95	0.01
	n	C(12)	IC(0)	PT	MSE	C(12)	IC(0)	PT	MSE
α	100	12.00	0.00	1.00	0.03	12.00	0.00	1.00	0.03
	500	12.00	0.00	1.00	0.00	12.00	0.00	1.00	0.00
	1000	12.00	0.00	1.00	0.00	12.00	0.00	1.00	0.00

C, average correct zeros; IC, average incorrect zeros; PT, the probability of choosing the true model; MSE, the average mean squared error.

Table C8: Estimation and inference metrics for MPR-SIC, BAMLSS, SPR-SIC and ALASSO-IC methods

MPR-SIC		$n = 100$				$n = 500$				$n = 1000$			
	θ	$\hat{\theta}$	SE	SEE	CP	$\hat{\theta}$	SE	SEE	CP	$\hat{\theta}$	SE	SEE	CP
β_0	0.0	0.01	0.19	0.15	0.87	-0.00	0.07	0.07	0.93	-0.00	0.05	0.05	0.94
β_1	1.0	1.00	0.12	0.10	0.88	1.00	0.05	0.04	0.94	1.00	0.03	0.03	0.95
β_2	0.5	0.44	0.27	0.12	0.72	0.50	0.08	0.07	0.94	0.50	0.05	0.05	0.95
β_3	0.5	0.49	0.15	0.11	0.88	0.50	0.05	0.05	0.94	0.50	0.04	0.04	0.96
β_4	1.0	1.00	0.11	0.09	0.90	1.00	0.04	0.04	0.95	1.00	0.03	0.03	0.95
β_5	0.5	0.49	0.13	0.09	0.88	0.50	0.05	0.04	0.94	0.50	0.03	0.03	0.95
β_6	1.0	1.05	0.27	0.15	0.71	1.00	0.08	0.07	0.94	1.00	0.05	0.05	0.93
α_0	0.0	-0.09	0.32	0.16	0.76	-0.02	0.08	0.06	0.91	-0.01	0.05	0.05	0.92

BAMLSS

	θ	$n = 100$ $\hat{\theta}$	SE	SEE	CP	$n = 500$ $\hat{\theta}$	SE	SEE	CP	$n = 1000$ $\hat{\theta}$	SE	SEE	CP
β_0	0.0	0.01	0.24	-	0.91	-0.01	0.09	-	0.95	0.01	0.06	-	0.96
β_1	1.0	1.00	0.13	-	0.90	1.01	0.05	-	0.94	1.00	0.03	-	0.92
β_2	0.5	0.49	0.22	-	0.89	0.50	0.08	-	0.94	0.50	0.05	-	0.94
β_3	0.5	0.50	0.13	-	0.92	0.50	0.05	-	0.95	0.50	0.04	-	0.95
β_4	1.0	0.99	0.11	-	0.95	1.00	0.04	-	0.97	1.00	0.03	-	0.93
β_5	0.5	0.50	0.13	-	0.91	0.50	0.05	-	0.90	0.50	0.03	-	0.92
β_6	1.0	1.00	0.30	-	0.89	1.00	0.10	-	0.91	0.99	0.06	-	0.96
α_0	0.0	0.01	0.41	-	0.92	-0.01	0.13	-	0.94	0.01	0.09	-	0.95

SPR-SIC

	θ	$n = 100$ $\hat{\theta}$	SE	SEE	CP	$n = 500$ $\hat{\theta}$	SE	SEE	CP	$n = 1000$ $\hat{\theta}$	SE	SEE	CP
β_0	0.0	0.06	0.18	0.16	0.92	0.01	0.07	0.07	0.93	0.01	0.05	0.05	0.94
β_1	1.0	0.98	0.11	0.11	0.92	1.00	0.05	0.05	0.95	1.00	0.03	0.03	0.95
β_2	0.5	0.41	0.24	0.15	0.81	0.48	0.08	0.08	0.91	0.48	0.05	0.05	0.93
β_3	0.5	0.43	0.15	0.12	0.85	0.48	0.05	0.05	0.94	0.49	0.04	0.04	0.94
β_4	1.0	0.98	0.11	0.10	0.94	0.99	0.04	0.05	0.95	1.00	0.03	0.03	0.95
β_5	0.5	0.45	0.13	0.10	0.88	0.49	0.05	0.05	0.92	0.49	0.03	0.03	0.94
β_6	1.0	1.04	0.24	0.17	0.80	1.01	0.08	0.08	0.92	1.01	0.06	0.05	0.93

ALASSO-IC

	θ	$n = 100$ $\hat{\theta}$	SE	SEE	CP	$n = 500$ $\hat{\theta}$	SE	SEE	CP	$n = 1000$ $\hat{\theta}$	SE	SEE	CP
β_0	0.0	-0.00	0.25	0.24	0.93	0.00	0.11	0.11	0.94	0.00	0.08	0.08	0.96
β_1	1.0	0.87	0.36	0.22	0.83	0.97	0.13	0.11	0.92	0.99	0.09	0.08	0.91
β_2	0.5	0.33	0.33	0.13	0.56	0.44	0.18	0.10	0.79	0.47	0.12	0.08	0.81
β_3	0.5	0.31	0.29	0.14	0.60	0.45	0.15	0.11	0.85	0.47	0.10	0.08	0.88
β_4	1.0	0.85	0.33	0.22	0.84	0.97	0.12	0.11	0.93	0.99	0.08	0.08	0.95
β_5	0.5	0.32	0.28	0.14	0.64	0.43	0.13	0.11	0.88	0.46	0.09	0.08	0.90
β_6	1.0	0.85	0.33	0.22	0.84	0.97	0.12	0.11	0.94	0.99	0.08	0.08	0.95

SE, standard deviation of estimates over 1000 replications; SEE, average of estimated standard errors over 1000 replications (200 replications for BAMLSS); CP, the empirical coverage probability of a nominal 95% confidence interval.

Appendix D Simulation Results: Normal Setting

This section contains additional simulation results for the MPR-SIC, BAMLSS, SPR-SIC and ALASSO-IC methods in a normal setting. Data are simulated from the normal MPR model, where X_4, X_5, X_7 and X_9 are Bernoulli(0.5) and the remainder are $N(0, 1)$. Note that fewer replicates for the BAMLSS procedure are used (200 replicates) due to the computational intensity of the method. The other approaches are averaged over 1000 replicates.

- Table D9 is analogous to Table 2 of the main paper, but showing the true values that are used to simulate data in a normal setting.
- Table D10 is analogous to Table 6 of the main paper, but showing the out-of-sample prediction coverage probabilities for the normal setting.
- Table D11 is analogous to Table 4 of the main paper, but showing the model selection metrics for the normal setting.
- Table D12 is analogous to Table 5 of the main paper, but showing the estimation and inference metrics for the normal setting.

Table D9: True parameter values

	X_0	X_1	X_2	X_3	$\boldsymbol{X_4}$	$\boldsymbol{X_5}$	X_6	$\boldsymbol{X_7}$	X_8	$\boldsymbol{X_9}$	X_{10}	X_{11}	X_{12}
β	0	1	0.5	0.5	1	0.5	1	0	0	0	0	0	0
α	0	0.5	1	0.5	1	0	0	0.5	1	0	0	0	0

Binary covariates indicated in bold.

Table D10: Simulation results: out-of-sample prediction coverage probabilities

	MPR-SIC			BAMLSS			SPR-SIC			ALASSO-IC		
n	100	500	1000	100	500	1000	100	500	1000	100	500	1000
Low	0.78	0.93	0.94	0.80	0.93	0.94	1.00	1.00	1.00	1.00	1.00	1.00
Medium	0.89	0.94	0.95	0.90	0.94	0.95	0.97	1.00	1.00	0.98	1.00	1.00
High	0.94	0.95	0.95	0.94	0.95	0.95	0.79	0.84	0.85	0.81	0.85	0.85
Overall	0.86	0.94	0.95	0.87	0.94	0.95	0.93	0.95	0.95	0.93	0.95	0.95

Variability categorized as low ($\sigma_i \leq 0.7$), medium ($\sigma_i \in (0.7, 1.5]$) and high ($\sigma_i > 1.5$). Out-of-sample coverage is calculated for a sample 20% the size of the original data.

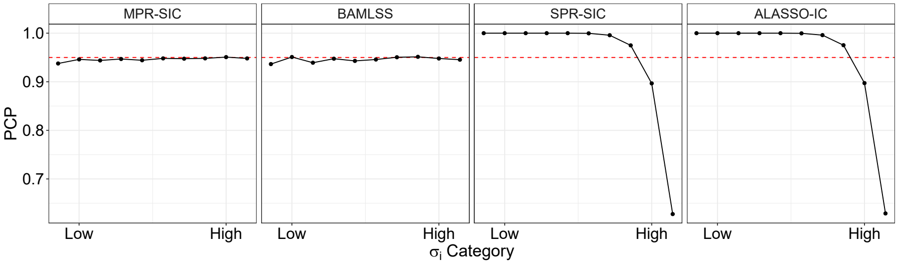

Fig. D1: Prediction coverage probabilities (PCPs) of observations split by variability σ_i. Solid black line indicates the coverage and the red dashed line is a reference line at 0.95.

Table D11: Simulation results: model selection metrics

		MPR-SIC				BAMLSS			
	n	C(6)	IC(0)	PT	MSE	C(6)	IC(0)	PT	MSE
β	100	5.27	0.00	0.50	0.05	5.63	0.00	0.71	0.08
	500	5.90	0.00	0.90	0.00	5.61	0.00	0.69	0.01
	1000	5.93	0.00	0.93	0.00	5.72	0.00	0.76	0.00
α	100	5.48	0.68	0.31	0.61	5.58	0.83	0.27	0.47
	500	5.93	0.00	0.93	0.04	5.70	0.00	0.74	0.06
	1000	5.94	0.00	0.94	0.02	5.71	0.00	0.73	0.03
		SPR-SIC				ALASSO-IC			
	n	C(6)	IC(0)	PT	MSE	C(6)	IC(0)	PT	MSE
β	100	5.65	1.49	0.16	0.91	5.38	1.31	0.14	0.86
	500	5.84	0.15	0.75	0.15	5.68	0.10	0.68	0.15
	1000	5.91	0.01	0.90	0.06	5.80	0.01	0.82	0.07
α	100	6.00	6.00	0.00	6.34	6.00	6.00	0.00	6.58
	500	6.00	6.00	0.00	6.83	6.00	6.00	0.00	6.89
	1000	6.00	6.00	0.00	6.90	6.00	6.00	0.00	6.93

C, average correct zeros; IC, average incorrect zeros; PT, the probability of choosing the true model; MSE, the average mean squared error.

Table D12: Estimation and inference metrics for MPR-SIC, BAMLSS, SPR-SIC and ALASSO-IC methods

MPR-SIC													
		$n = 100$				$n = 500$				$n = 1000$			
	θ	$\hat{\theta}$	SE	SEE	CP	$\hat{\theta}$	SE	SEE	CP	$\hat{\theta}$	SE	SEE	CP
β_0	0.0	-0.00	0.12	0.08	0.84	0.00	0.04	0.04	0.93	0.00	0.03	0.03	0.95
β_1	1.0	1.00	0.07	0.05	0.82	1.00	0.02	0.02	0.93	1.00	0.01	0.01	0.95
β_2	0.5	0.49	0.08	0.05	0.81	0.50	0.02	0.02	0.93	0.50	0.01	0.01	0.96
β_3	0.5	0.50	0.07	0.05	0.80	0.50	0.02	0.02	0.94	0.50	0.01	0.01	0.95
β_4	1.0	1.00	0.09	0.06	0.82	1.00	0.03	0.03	0.94	1.00	0.02	0.02	0.95
β_5	0.5	0.50	0.07	0.04	0.80	0.50	0.02	0.02	0.94	0.50	0.01	0.01	0.93
β_6	1.0	1.00	0.07	0.05	0.79	1.00	0.02	0.02	0.94	1.00	0.01	0.01	0.94
α_0	0.0	-0.22	0.18	0.15	0.66	-0.03	0.07	0.06	0.91	-0.02	0.05	0.04	0.94
α_1	0.5	0.49	0.31	0.13	0.68	0.51	0.07	0.07	0.94	0.50	0.05	0.05	0.95
α_2	1.0	1.12	0.25	0.17	0.81	1.01	0.07	0.07	0.95	1.00	0.05	0.05	0.95
α_3	0.5	0.49	0.31	0.13	0.67	0.51	0.07	0.07	0.94	0.50	0.04	0.05	0.95
α_4	1.0	1.09	0.21	0.16	0.84	1.01	0.07	0.06	0.93	1.00	0.05	0.05	0.95
α_7	0.5	0.49	0.30	0.13	0.67	0.51	0.07	0.06	0.94	0.50	0.05	0.05	0.94
α_8	1.0	1.13	0.24	0.17	0.81	1.02	0.07	0.07	0.94	1.01	0.05	0.05	0.95
BAMLSS													
		$n = 100$				$n = 500$				$n = 1000$			
	θ	$\hat{\theta}$	SE	SEE	CP	$\hat{\theta}$	SE	SEE	CP	$\hat{\theta}$	SE	SEE	CP
β_0	0.0	-0.01	0.13	-	0.90	0.00	0.05	-	0.94	0.00	0.03	-	0.95
β_1	1.0	0.99	0.07	-	0.93	1.00	0.02	-	0.93	1.00	0.01	-	0.95
β_2	0.5	0.49	0.08	-	0.92	0.50	0.02	-	0.92	0.50	0.01	-	0.94
β_3	0.5	0.50	0.07	-	0.92	0.50	0.02	-	0.94	0.50	0.01	-	0.96
β_4	1.0	1.00	0.09	-	0.90	1.00	0.03	-	0.91	1.00	0.02	-	0.94
β_5	0.5	0.51	0.07	-	0.91	0.50	0.02	-	0.88	0.50	0.01	-	0.95
β_6	1.0	1.00	0.07	-	0.92	1.00	0.02	-	0.95	1.00	0.01	-	0.94
α_0	0.0	-0.03	0.17	-	0.94	-0.00	0.07	-	0.95	-0.00	0.04	-	0.96
α_1	0.5	0.50	0.22	-	0.92	0.51	0.07	-	0.92	0.50	0.05	-	0.92
α_2	1.0	1.03	0.21	-	0.95	1.00	0.07	-	0.94	1.00	0.04	-	0.95
α_3	0.5	0.51	0.19	-	0.97	0.50	0.07	-	0.92	0.50	0.05	-	0.94
α_4	1.0	1.00	0.20	-	0.94	1.00	0.06	-	0.96	1.00	0.05	-	0.95
α_7	0.5	0.51	0.21	-	0.90	0.51	0.07	-	0.93	0.50	0.04	-	0.96
α_8	1.0	1.00	0.20	-	0.94	1.00	0.06	-	0.96	1.00	0.05	-	0.96
SPR-SIC													
		$n = 100$				$n = 500$				$n = 1000$			
	θ	$\hat{\theta}$	SE	SEE	CP	$\hat{\theta}$	SE	SEE	CP	$\hat{\theta}$	SE	SEE	CP
β_0	0.0	-0.01	0.25	0.23	0.92	0.00	0.11	0.11	0.94	0.00	0.08	0.08	0.96
β_1	1.0	0.99	0.36	0.21	0.87	1.00	0.12	0.11	0.92	1.00	0.09	0.08	0.92
β_2	0.5	0.39	0.40	0.12	0.47	0.49	0.18	0.10	0.83	0.51	0.11	0.08	0.84
β_3	0.5	0.36	0.36	0.11	0.51	0.50	0.16	0.10	0.89	0.50	0.09	0.08	0.93
β_4	1.0	0.97	0.31	0.21	0.89	1.00	0.11	0.11	0.94	1.00	0.07	0.08	0.95
β_5	0.5	0.39	0.35	0.12	0.56	0.49	0.14	0.10	0.93	0.50	0.08	0.08	0.95
β_6	1.0	0.96	0.34	0.21	0.88	1.00	0.11	0.11	0.94	1.00	0.08	0.08	0.97

ALASSO-IC

		$n = 100$				$n = 500$				$n = 1000$			
	θ	$\hat{\theta}$	SE	SEE	CP	$\hat{\theta}$	SE	SEE	CP	$\hat{\theta}$	SE	SEE	CP
β_0	0.0	-0.00	0.25	0.24	0.93	0.00	0.11	0.11	0.94	0.00	0.08	0.08	0.96
β_1	1.0	0.87	0.36	0.22	0.83	0.97	0.13	0.11	0.92	0.99	0.09	0.08	0.91
β_2	0.5	0.33	0.33	0.13	0.56	0.44	0.18	0.10	0.79	0.47	0.12	0.08	0.81
β_3	0.5	0.31	0.29	0.14	0.60	0.45	0.15	0.11	0.85	0.47	0.10	0.08	0.88
β_4	1.0	0.85	0.33	0.22	0.84	0.97	0.12	0.11	0.93	0.99	0.08	0.08	0.95
β_5	0.5	0.32	0.28	0.14	0.64	0.43	0.13	0.11	0.88	0.46	0.09	0.08	0.90
β_6	1.0	0.85	0.33	0.22	0.84	0.97	0.12	0.11	0.94	0.99	0.08	0.08	0.95

SE, standard deviation of estimates over 1000 replications; SEE, average of estimated standard errors over 1000 replications (200 replications for BAMLSS); CP, the empirical coverage probability of a nominal 95% confidence interval.

Appendix E Simulation Results: Multiparameter Setting

This section displays additional simulation results for data simulated using the normal MPR model. Table E13 is analogous to Table 5 of the main paper, but showing estimation and inference metrics for the BAMLSS, SPR-SIC and ALASSO-IC methods.

Table E13: Estimation and inference metrics for BAMLSS, SPR-SIC and ALASSO-IC methods

BAMLSS

		$n = 100$				$n = 500$				$n = 1000$			
	θ	$\hat{\theta}$	SE	SEE	CP	$\hat{\theta}$	SE	SEE	CP	$\hat{\theta}$	SE	SEE	CP
β_0	0.0	-0.00	0.25	-	0.90	0.00	0.08	-	0.92	-0.00	0.05	-	0.94
β_1	1.0	1.01	0.15	-	0.92	1.00	0.05	-	0.94	1.00	0.03	-	0.95
β_2	0.5	0.49	0.18	-	0.93	0.50	0.05	-	0.95	0.50	0.03	-	0.96
β_3	0.5	0.50	0.11	-	0.93	0.50	0.03	-	0.94	0.50	0.02	-	0.95
β_4	1.0	1.01	0.12	-	0.91	1.00	0.03	-	0.95	1.00	0.02	-	0.95
β_5	0.5	0.50	0.11	-	0.93	0.50	0.03	-	0.95	0.50	0.02	-	0.95
β_6	1.0	1.01	0.23	-	0.94	1.00	0.07	-	0.94	1.00	0.04	-	0.96
α_0	0.0	-0.03	0.40	-	0.92	0.02	0.17	-	0.90	0.04	0.15	-	0.86
α_1	0.5	0.50	0.21	-	0.92	0.49	0.08	-	0.89	0.49	0.06	-	0.86
α_2	1.0	1.04	0.35	-	0.92	0.97	0.15	-	0.90	0.95	0.15	-	0.84
α_3	0.5	0.50	0.25	-	0.93	0.49	0.09	-	0.90	0.48	0.07	-	0.85
α_4	1.0	1.01	0.20	-	0.94	0.98	0.10	-	0.91	0.97	0.10	-	0.85
α_7	0.5	0.52	0.21	-	0.94	0.49	0.08	-	0.88	0.49	0.06	-	0.86
α_8	1.0	1.01	0.21	-	0.94	0.99	0.10	-	0.91	0.97	0.10	-	0.86

SPR-SIC

		$n = 100$				$n = 500$				$n = 1000$			
	θ	$\hat{\theta}$	SE	SEE	CP	$\hat{\theta}$	SE	SEE	CP	$\hat{\theta}$	SE	SEE	CP
β_0	0.0	0.28	0.78	0.45	0.72	0.10	0.37	0.24	0.83	0.01	0.26	0.18	0.88
β_1	1.0	0.90	0.76	0.23	0.59	0.98	0.38	0.16	0.69	1.01	0.27	0.12	0.69
β_2	0.5	0.51	0.78	0.12	0.09	0.36	0.52	0.08	0.21	0.35	0.37	0.09	0.44
β_3	0.5	0.18	0.38	0.06	0.17	0.34	0.30	0.10	0.57	0.46	0.19	0.12	0.88
β_4	1.0	0.90	0.63	0.24	0.68	0.99	0.27	0.16	0.84	1.00	0.16	0.12	0.85
β_5	0.5	0.25	0.41	0.09	0.28	0.41	0.28	0.11	0.68	0.48	0.15	0.11	0.92
β_6	1.0	0.76	0.82	0.16	0.33	1.06	0.53	0.16	0.46	1.11	0.33	0.15	0.54

ALASSO-IC

		$n = 100$				$n = 500$				$n = 1000$			
	θ	$\hat{\theta}$	SE	SEE	CP	$\hat{\theta}$	SE	SEE	CP	$\hat{\theta}$	SE	SEE	CP
β_0	0.0	0.45	0.70	0.47	0.68	0.20	0.37	0.25	0.79	0.09	0.26	0.18	0.85
β_1	1.0	0.74	0.67	0.24	0.59	0.91	0.38	0.16	0.66	0.97	0.28	0.12	0.66
β_2	0.5	0.38	0.60	0.16	0.26	0.38	0.41	0.15	0.49	0.37	0.31	0.13	0.64
β_3	0.5	0.14	0.29	0.09	0.23	0.29	0.23	0.12	0.66	0.38	0.16	0.12	0.85
β_4	1.0	0.72	0.56	0.25	0.67	0.90	0.29	0.16	0.78	0.96	0.17	0.12	0.81
β_5	0.5	0.18	0.30	0.10	0.32	0.34	0.23	0.12	0.72	0.41	0.15	0.11	0.85
β_6	1.0	0.71	0.66	0.25	0.54	0.97	0.42	0.21	0.71	1.05	0.27	0.17	0.73

SE, standard deviation of estimates over 1000 replications; SEE, average of estimated standard errors over 1000 replications; CP, the empirical coverage probability of a nominal 95% confidence interval.

Appendix F Simulation Results: Different Effect Sizes

This section contains additional simulation results for the MPR-SIC method, with different effect sizes.

F.1 Greater Location Effects

- Table F14 is analogous to Table 2 of the main paper, but showing the new true values that are used to simulate data. The location component has greater effect sizes, resulting in a mean-driven problem.
- Table F15 is analogous to Table 4 of the main paper, but showing the model selection metrics for the setting with greater location effects. The results are comparable to the setting where the location and dispersion effects are on the same scale.
- Table F16 is analogous to Table 5 of the main paper, but gives the estimation and inference metrics for the setting with greater location effects. The results are comparable to the setting where the location and dispersion effects are on the same scale.

Table F14: True parameter values for the setting with greater location effects

		E	M	B	N	N	M	N	N	M	B	E	M
	X_0	X_1	X_2	X_3	X_4	X_5	X_6	X_7	X_8	X_9	X_{10}	X_{11}	X_{12}
β	0	10	5	5	10	5	10	0	0	0	0	0	0
α	0	0.5	1	0.5	1	0	0	0.5	1	0	0	0	0

E = Exponential, B = Bernoulli, N = independent normal,
M = multivariate normal (correlated).

Table F15: Simulation results: model selection metrics for the setting with greater location effects

		MPR-SIC			
	n	C(6)	IC(0)	PT	MSE
β	100	5.28	0.00	0.53	0.13
	500	5.91	0.00	0.91	0.01
	1000	5.95	0.00	0.95	0.00
α	100	5.54	0.75	0.34	0.61
	500	5.92	0.00	0.93	0.03
	1000	5.95	0.00	0.95	0.02

C, average correct zeros; IC, average incorrect zeros; PT, the probability of choosing the true model; MSE, the average mean squared error.

Table F16: Simulation results: estimation and inference metrics for the setting with greater location effects

MPR-SIC		$n=100$				$n=500$				$n=1000$			
	θ	$\hat{\theta}$	SE	SEE	CP	$\hat{\theta}$	SE	SEE	CP	$\hat{\theta}$	SE	SEE	CP
β_0	0.00	0.01	0.21	0.13	0.79	-0.00	0.06	0.05	0.94	-0.00	0.04	0.04	0.94
β_1	10.0	10.0	0.15	0.10	0.78	10.0	0.04	0.04	0.94	10.0	0.03	0.03	0.96
β_2	5.00	5.01	0.18	0.12	0.80	5.00	0.05	0.05	0.92	5.00	0.03	0.03	0.94
β_3	5.00	4.99	0.10	0.07	0.80	5.00	0.03	0.03	0.94	5.00	0.02	0.02	0.94
β_4	10.0	10.0	0.11	0.07	0.81	10.0	0.03	0.03	0.94	10.0	0.02	0.02	0.93
β_5	5.00	5.01	0.10	0.07	0.79	5.00	0.03	0.03	0.93	5.00	0.02	0.02	0.95
β_6	10.0	9.99	0.20	0.12	0.77	10.0	0.05	0.05	0.92	10.0	0.03	0.03	0.94
α_0	0.00	-0.17	0.43	0.23	0.66	-0.03	0.11	0.10	0.90	-0.02	0.07	0.07	0.92
α_1	0.50	0.45	0.28	0.13	0.72	0.50	0.07	0.07	0.93	0.50	0.05	0.05	0.94
α_2	1.00	1.07	0.36	0.17	0.78	1.01	0.07	0.07	0.92	1.01	0.05	0.05	0.93
α_3	0.50	0.51	0.36	0.14	0.59	0.51	0.08	0.08	0.95	0.51	0.05	0.05	0.94
α_4	1.00	1.11	0.24	0.17	0.82	1.01	0.06	0.07	0.95	1.01	0.05	0.05	0.94
α_7	0.50	0.51	0.29	0.14	0.72	0.51	0.06	0.07	0.96	0.50	0.05	0.05	0.94
α_8	1.00	1.11	0.24	0.17	0.82	1.01	0.07	0.07	0.93	1.01	0.05	0.05	0.94

SE, standard deviation of estimates over 1000 replications; SEE, average of estimated standard errors over 1000 replications; CP, the empirical coverage probability of a nominal 95% confidence interval.

F.2 Greater Dispersion Effects

- Table F17 is analogous to Table 2 of the main paper, but showing the new true values that are used to simulate data. The dispersion component has greater effect sizes, resulting in a dispersion-driven problem.
- Table F18 is analogous to Table 4 of the main paper, but showing the model selection metrics for the setting with greater location effects. This dispersion-driven problem results in poor variable selection metrics for the location component of the model.
- Table F19 is analogous to Table 5 of the main paper, but gives the estimation and inference metrics for the setting with greater location effects. For $n = 100$, the coverage of the location parameters is poor, but improves as the sample size increases.

Table F17: True parameter values for the setting with greater dispersion effects

		E	M	B	N	N	M	N	N	M	B	E	M
	X_0	X_1	X_2	X_3	X_4	X_5	X_6	X_7	X_8	X_9	X_{10}	X_{11}	X_{12}
β	0	1	0.5	0.5	1	0.5	1	0	0	0	0	0	0
α	0	1.5	3	1.5	3	0	0	1.5	3	0	0	0	0

E = Exponential, B = Bernoulli, N = independent normal,
M = multivariate normal (correlated).

Table F18: Simulation results: model selection metrics for the setting with greater dispersion effects

		MPR-SIC			
	n	C(6)	IC(0)	PT	MSE
β	100	5.15	0.00	0.52	0.02
	500	4.42	0.00	0.26	0.00
	1000	2.97	0.00	0.04	0.00
α	100	5.60	0.00	0.69	0.50
	500	5.93	0.00	0.93	0.04
	1000	5.94	0.00	0.94	0.02

C, average correct zeros; IC, average incorrect zeros; PT, the probability of choosing the true model; MSE, the average mean squared error.

Table F19: Simulation results: estimation and inference metrics for the setting with greater dispersion effects

MPR-SIC		$n = 100$				$n = 500$				$n = 1000$			
	θ	$\hat{\theta}$	SE	SEE	CP	$\hat{\theta}$	SE	SEE	CP	$\hat{\theta}$	SE	SEE	CP
β_0	0.0	0.00	0.07	0.03	0.70	-0.00	0.01	0.00	0.92	0.00	0.00	0.00	0.92
β_1	1.0	1.00	0.05	0.02	0.70	1.00	0.00	0.00	0.92	1.00	0.00	0.00	0.94
β_2	0.5	0.50	0.05	0.02	0.75	0.50	0.00	0.00	0.92	0.50	0.00	0.00	0.94
β_3	0.5	0.50	0.03	0.01	0.72	0.50	0.00	0.00	0.92	0.50	0.00	0.00	0.93
β_4	1.0	1.00	0.04	0.02	0.70	1.00	0.00	0.00	0.93	1.00	0.00	0.00	0.93
β_5	0.5	0.50	0.03	0.01	0.73	0.50	0.00	0.00	0.92	0.50	0.00	0.00	0.93
β_6	1.0	1.00	0.06	0.02	0.70	1.00	0.00	0.00	0.90	1.00	0.00	0.00	0.94
α_0	0.0	-0.35	0.33	0.25	0.67	-0.04	0.11	0.10	0.91	-0.02	0.07	0.07	0.92
α_1	1.5	1.54	0.20	0.17	0.91	1.50	0.07	0.07	0.93	1.50	0.05	0.05	0.94
α_2	3.0	3.16	0.27	0.18	0.79	3.02	0.07	0.07	0.92	3.01	0.05	0.05	0.93
α_3	1.5	1.63	0.25	0.19	0.83	1.51	0.08	0.07	0.94	1.51	0.05	0.05	0.94
α_4	3.0	3.16	0.22	0.17	0.82	3.02	0.06	0.07	0.94	3.01	0.05	0.05	0.94
α_7	1.5	1.59	0.20	0.16	0.88	1.51	0.06	0.07	0.95	1.50	0.05	0.05	0.94
α_8	3.0	3.16	0.22	0.17	0.80	3.02	0.07	0.07	0.92	3.01	0.05	0.05	0.94

SE, standard deviation of estimates over 1000 replications; SEE, average of estimated standard errors over 1000 replications; CP, the empirical coverage probability of a nominal 95% confidence interval.

Appendix G Simulation Results: Imbalance of Active Sets

This section contains additional simulation results for the MPR-SIC method, with changes in the cardinality of the active sets.

G.1 Location Component

- Table G20 is analogous to Table 2 of the main paper, but showing the new true values that are used to simulate data. The cardinality of the active set for the location component is greater than the dispersion component.
- Table G21 is analogous to Table 4 of the main paper, but showing the model selection metrics for the setting where the active set of the location component contains more parameters than the dispersion component. Performance is comparable with the performance when the cardinality of the active sets are identical.
- Table G22 is analogous to Table 5 of the main paper, but gives the estimation and inference metrics for the setting where the cardinality of the active set for the location component is greater than the dispersion component. Performance is comparable with the performance when the cardinality of the active sets are identical.

Table G20: True parameter values for the setting where the cardinality of the active set is greater for the location component

	E	M	B	N	N	M	N	N	M	B	E	M
	X_0	X_1	X_2	X_3	X_4	X_5	X_6	X_7	X_8	X_9	X_{10}	X_{11}	X_{12}
β	0	1	0.5	0.5	1	0.5	1	0	0	0	0	0	0
α	0	0	1	0	1	0	0	0	1	0	0	0	0

E = Exponential, B = Bernoulli, N = independent normal,
M = multivariate normal (correlated).

Table G21: Simulation results: model selection metrics for the setting where the cardinality of the active set is greater for the location component

		MPR-SIC			
	n	C(6)	IC(0)	PT	MSE
β	100	5.40	0.03	0.57	0.06
	500	5.91	0.00	0.92	0.01
	1000	5.95	0.00	0.95	0.00
	n	C(9)	IC(0)	PT	MSE
α	100	8.34	0.05	0.55	0.38
	500	8.89	0.00	0.90	0.02
	1000	8.93	0.00	0.93	0.01

C, average correct zeros; IC, average incorrect zeros; PT, the probability of choosing the true model; MSE, the average mean squared error.

Table G22: Simulation results: estimation and inference metrics for the setting where the cardinality of the active set is greater for the location component

MPR-SIC		$n = 100$				$n = 500$				$n = 1000$			
	θ	$\hat{\theta}$	SE	SEE	CP	$\hat{\theta}$	SE	SEE	CP	$\hat{\theta}$	SE	SEE	CP
β_0	0.0	0.01	0.16	0.11	0.81	-0.00	0.05	0.05	0.94	0.00	0.03	0.03	0.94
β_1	1.0	1.00	0.08	0.06	0.84	1.00	0.02	0.02	0.95	1.00	0.02	0.02	0.95
β_2	0.5	0.50	0.14	0.09	0.82	0.50	0.04	0.04	0.92	0.50	0.03	0.03	0.95
β_3	0.5	0.50	0.09	0.06	0.84	0.50	0.03	0.03	0.95	0.50	0.02	0.02	0.94
β_4	1.0	1.00	0.08	0.06	0.84	1.00	0.02	0.02	0.94	1.00	0.02	0.02	0.94
β_5	0.5	0.50	0.07	0.05	0.85	0.50	0.02	0.02	0.93	0.50	0.02	0.02	0.95
β_6	1.0	1.00	0.15	0.09	0.80	1.00	0.04	0.04	0.93	1.00	0.03	0.03	0.93
α_0	0.0	-0.18	0.28	0.16	0.68	-0.02	0.08	0.06	0.90	-0.01	0.05	0.05	0.93
α_2	1.0	1.06	0.34	0.17	0.81	1.01	0.07	0.07	0.93	1.01	0.05	0.05	0.93
α_4	1.0	1.10	0.22	0.17	0.84	1.01	0.06	0.07	0.95	1.01	0.05	0.05	0.95
α_8	1.0	1.11	0.22	0.17	0.85	1.01	0.07	0.07	0.93	1.01	0.05	0.05	0.93

SE, standard deviation of estimates over 1000 replications; SEE, average of estimated standard errors over 1000 replications; CP, the empirical coverage probability of a nominal 95% confidence interval.

G.2 Dispersion Component

- Table G23 is analogous to Table 2 of the main paper, but showing the new true values that are used to simulate data. The cardinality of the active set for the dispersion component is greater than the location component.
- Table G24 is analogous to Table 4 of the main paper, but showing the model selection metrics for the setting where the active set of the dispersion component contains more parameters than the location component. Performance is comparable with the performance when the cardinality of the active sets are identical.
- Table G25 is analogous to Table 5 of the main paper, but gives the estimation and inference metrics for the setting where the cardinality of the active set for the dispersion component is greater than the location component. Performance is comparable with the performance when the cardinality of the active sets are identical.

Table G23: True parameter values for the setting where the cardinality of the active set is greater for the dispersion component

	X_0	E<br>X_1	M<br>X_2	B<br>X_3	N<br>X_4	N<br>X_5	M<br>X_6	N<br>X_7	N<br>X_8	M<br>X_9	B<br>X_{10}	E<br>X_{11}	M<br>X_{12}
β	0	1	0	0	1	0	1	0	0	0	0	0	0
α	0	0.5	1	0.5	1	0	0	0.5	1	0	0	0	0

E = Exponential, B = Bernoulli, N = independent normal,
M = multivariate normal (correlated).

Table G24: Simulation results: model selection metrics for the setting where the cardinality of the active set is greater for the dispersion component

		MPR-SIC			
	n	C(9)	IC(0)	PT	MSE
β	100	8.04	0.00	0.45	0.10
	500	8.87	0.00	0.88	0.01
	1000	8.93	0.00	0.93	0.00
	n	C(6)	IC(0)	PT	MSE
α	100	5.57	0.73	0.34	0.53
	500	5.93	0.00	0.93	0.03
	1000	5.95	0.00	0.95	0.02

C, average correct zeros; IC, average incorrect zeros; PT, the probability of choosing the true model; MSE, the average mean squared error.

Table G25: Simulation results: estimation and inference metrics for the setting where the cardinality of the active set is greater for the dispersion component

MPR-SIC		$n = 100$				$n = 500$				$n = 1000$			
	θ	$\hat{\theta}$	SE	SEE	CP	$\hat{\theta}$	SE	SEE	CP	$\hat{\theta}$	SE	SEE	CP
β_0	0.0	0.01	0.19	0.12	0.77	0.00	0.06	0.05	0.93	-0.00	0.04	0.04	0.94
β_1	1.0	1.00	0.14	0.09	0.79	1.00	0.04	0.04	0.94	1.00	0.03	0.03	0.96
β_4	1.0	1.00	0.10	0.07	0.81	1.00	0.03	0.03	0.95	1.00	0.02	0.02	0.93
β_6	1.0	0.99	0.16	0.07	0.74	1.00	0.04	0.03	0.93	1.00	0.02	0.02	0.92
α_0	0.0	-0.12	0.42	0.22	0.68	-0.02	0.11	0.10	0.91	-0.01	0.07	0.07	0.92
α_1	0.5	0.44	0.27	0.13	0.73	0.50	0.07	0.07	0.93	0.50	0.05	0.05	0.95
α_2	1.0	1.05	0.34	0.16	0.80	1.01	0.07	0.07	0.93	1.00	0.05	0.05	0.93
α_3	0.5	0.49	0.35	0.13	0.61	0.51	0.08	0.07	0.95	0.50	0.05	0.05	0.94
α_4	1.0	1.08	0.23	0.17	0.84	1.01	0.06	0.07	0.95	1.00	0.05	0.05	0.94
α_7	0.5	0.50	0.28	0.13	0.74	0.50	0.06	0.07	0.96	0.50	0.05	0.05	0.94
α_8	1.0	1.08	0.22	0.17	0.83	1.01	0.07	0.07	0.93	1.00	0.05	0.05	0.94

SE, standard deviation of estimates over 1000 replications; SEE, average of estimated standard errors over 1000 replications; CP, the empirical coverage probability of a nominal 95% confidence interval.

Appendix H Simulation Results: Additional Covariates

This section contains additional simulation results for the MPR-SIC method, with additional covariates.

H.1 Additional Noise

- Table H26 is analogous to Table 2 of the main paper, but showing the new true values that are used to simulate data with additional covariates.
- Table H27 is analogous to Table 4 of the main paper, but showing the model selection metrics for the scenario with additional covariates.
- Table H28 is analogous to Table 5 of the main paper, but gives the estimation and inference metrics for the scenario with additional covariates.

Table H26: True parameter values for the scenario with additional covariates

		E	M	B	N	N	M	N	N	M	B	E	M
	X_0	X_1	X_2	X_3	X_4	X_5	X_6	X_7	X_8	X_9	X_{10}	X_{11}	X_{12}
β	0	1	0	0	1	0	1	0	0	0	0	0	0
α	0	0.5	1	0.5	1	0	0	0.5	1	0	0	0	0
		N	N	N	N	N	N	N	N	N	N	N	N
		X_{13}	X_{14}	X_{15}	X_{16}	X_{17}	X_{18}	X_{19}	X_{20}	X_{21}	X_{22}	X_{23}	X_{24}
β		0	0	0	0	0	0	0	0	0	0	0	0
α		0	0	0	0	0	0	0	0	0	0	0	0

E = Exponential, B = Bernoulli, N = independent normal,
M = multivariate normal (correlated).

Table H27: Simulation results: model selection metrics for the scenario with additional covariates

		MPR-SIC			
	n	C(18)	IC(0)	PT	MSE
β	500	17.64	0.00	0.71	0.01
	1000	17.82	0.00	0.84	0.00
	2000	17.87	0.00	0.88	0.00
α	500	17.72	0.00	0.76	0.04
	100	17.81	0.00	0.82	0.02
	2000	17.90	0.00	0.91	0.01

C, average correct zeros; IC, average incorrect zeros; PT, the probability of choosing the true model; MSE, the average mean squared error.

Table H28: Simulation results: estimation and inference metrics for the scenario with additional covariates

MPR-SIC		$n = 500$				$n = 1000$				$n = 2000$			
	θ	$\hat{\theta}$	SE	SEE	CP	$\hat{\theta}$	SE	SEE	CP	$\hat{\theta}$	SE	SEE	CP
β_0	0.0	-0.00	0.06	0.06	0.92	0.00	0.04	0.04	0.94	-0.00	0.03	0.03	0.95
β_1	1.0	1.00	0.04	0.04	0.93	1.00	0.03	0.03	0.94	1.00	0.02	0.02	0.95
β_2	0.5	0.50	0.05	0.05	0.92	0.50	0.03	0.03	0.95	0.50	0.02	0.02	0.94
β_3	0.5	0.50	0.03	0.03	0.93	0.50	0.02	0.02	0.94	0.50	0.01	0.01	0.95
β_4	1.0	1.00	0.03	0.03	0.92	1.00	0.02	0.02	0.94	1.00	0.01	0.01	0.95
β_5	0.5	0.50	0.03	0.03	0.93	0.50	0.02	0.02	0.94	0.50	0.01	0.01	0.95
β_6	1.0	1.00	0.06	0.05	0.91	1.00	0.03	0.03	0.94	1.00	0.02	0.02	0.93
α_0	0.0	-0.04	0.11	0.10	0.91	-0.02	0.07	0.07	0.95	-0.01	0.05	0.05	0.95
α_1	0.5	0.50	0.07	0.07	0.95	0.50	0.05	0.05	0.94	0.50	0.03	0.03	0.95
α_2	1.0	1.02	0.08	0.07	0.92	1.01	0.05	0.05	0.93	1.00	0.03	0.03	0.94
α_3	0.5	0.52	0.08	0.08	0.95	0.51	0.05	0.05	0.94	0.50	0.04	0.04	0.95
α_4	1.0	1.02	0.07	0.07	0.93	1.01	0.05	0.05	0.95	1.00	0.03	0.03	0.95
α_7	0.5	0.51	0.07	0.07	0.95	0.50	0.05	0.05	0.94	0.50	0.03	0.03	0.96
α_8	1.0	1.02	0.07	0.07	0.93	1.01	0.05	0.05	0.94	1.00	0.03	0.03	0.95

SE, standard deviation of estimates over 1000 replications; SEE, average of estimated standard errors over 1000 replications; CP, the empirical coverage probability of a nominal 95% confidence interval.

H.2 Repeated Covariates

- Table H29 is analogous to Table 2 of the main paper, but showing the new true values that are used to simulate data with additional covariates.
- Table H30 is analogous to Table 4 of the main paper, but showing the model selection metrics for the scenario with additional covariates.
- Table H31 is analogous to Table 5 of the main paper, but gives the estimation and inference metrics for the scenario with additional covariates.

Table H29: True parameter values for the scenario with additional covariates

		E	M_1	B	N	N	M_1	N	N	M_1	B	E	M_1
	X_0	X_1	X_2	X_3	X_4	X_5	X_6	X_7	X_8	X_9	X_{10}	X_{11}	X_{12}
β	0	1	0	0	1	0	1	0	0	0	0	0	0
α	0	0.5	1	0.5	1	0	0	0.5	1	0	0	0	0
		E	M_2	B	N	N	M_2	N	N	M_2	B	E	M_2
		X_{13}	X_{14}	X_{15}	X_{16}	X_{17}	X_{18}	X_{19}	X_{20}	X_{21}	X_{22}	X_{23}	X_{24}
β		1	0	0	1	0	1	0	0	0	0	0	0
α		0.5	1	0.5	1	0	0	0.5	1	0	0	0	0

E = Exponential, B = Bernoulli, N = independent normal,
M_1 = multivariate normal (first set of correlated covariates),
M_2 = multivariate normal (second set of correlated covariates).

Table H30: Simulation results: model selection metrics for the scenario with additional covariates

		MPR-SIC			
	n	C(12)	IC(0)	PT	MSE
β	500	11.73	0.00	0.77	0.01
	1000	11.85	0.00	0.86	0.00
	2000	11.91	0.00	0.92	0.00
α	500	11.78	0.00	0.81	0.08
	100	11.90	0.00	0.91	0.03
	2000	11.93	0.00	0.94	0.01

C, average correct zeros; IC, average incorrect zeros; PT, the probability of choosing the true model; MSE, the average mean squared error.

Table H31: Simulation results: estimation and inference metrics for the scenario with additional covariates

MPR-SIC		n = 500				n = 1000				n = 2000			
	θ	$\hat{\theta}$	SE	SEE	CP	$\hat{\theta}$	SE	SEE	CP	$\hat{\theta}$	SE	SEE	CP
β_0	0.0	0.00	0.06	0.05	0.92	-0.00	0.04	0.04	0.92	0.00	0.03	0.02	0.94
β_1	1.0	1.00	0.04	0.03	0.93	1.00	0.02	0.02	0.93	1.00	0.01	0.01	0.93
β_2	0.5	0.50	0.04	0.04	0.92	0.50	0.03	0.02	0.93	0.50	0.02	0.02	0.94
β_3	0.5	0.50	0.03	0.02	0.90	0.50	0.02	0.01	0.94	0.50	0.01	0.01	0.93
β_4	1.0	1.00	0.03	0.02	0.92	1.00	0.02	0.02	0.94	1.00	0.01	0.01	0.94
β_5	0.5	0.50	0.03	0.02	0.94	0.50	0.02	0.01	0.93	0.50	0.01	0.01	0.95
β_6	1.0	1.00	0.05	0.04	0.91	1.00	0.03	0.02	0.92	1.00	0.02	0.02	0.94
β_{13}	1.0	1.00	0.04	0.03	0.92	1.00	0.02	0.02	0.92	1.00	0.01	0.01	0.95
β_{14}	0.5	0.50	0.05	0.04	0.92	0.50	0.03	0.03	0.93	0.50	0.02	0.02	0.93
β_{15}	0.5	0.50	0.03	0.02	0.90	0.50	0.02	0.01	0.94	0.50	0.01	0.01	0.95
β_{16}	1.0	1.00	0.03	0.02	0.91	1.00	0.02	0.02	0.93	1.00	0.01	0.01	0.95
β_{17}	0.5	0.50	0.03	0.02	0.92	0.50	0.02	0.01	0.93	0.50	0.01	0.01	0.94
β_{18}	1.0	1.00	0.05	0.04	0.92	1.00	0.03	0.02	0.92	1.00	0.02	0.02	0.94
α_0	0.0	-0.08	0.14	0.13	0.89	-0.04	0.09	0.09	0.91	-0.02	0.07	0.06	0.91
α_1	0.5	0.50	0.07	0.07	0.94	0.50	0.05	0.05	0.95	0.50	0.03	0.03	0.94
α_2	1.0	1.02	0.08	0.07	0.92	1.01	0.05	0.05	0.94	1.01	0.03	0.03	0.94
α_3	0.5	0.52	0.08	0.08	0.95	0.51	0.05	0.05	0.93	0.50	0.04	0.04	0.95
α_4	1.0	1.02	0.07	0.07	0.92	1.01	0.05	0.05	0.94	1.00	0.03	0.03	0.94
α_7	0.5	0.51	0.07	0.07	0.94	0.51	0.05	0.05	0.95	0.50	0.03	0.03	0.95
α_8	1.0	1.03	0.07	0.07	0.92	1.01	0.05	0.05	0.95	1.01	0.03	0.03	0.95
α_{13}	0.5	0.50	0.07	0.07	0.94	0.50	0.04	0.05	0.96	0.50	0.03	0.03	0.95
α_{14}	1.0	1.02	0.08	0.07	0.90	1.01	0.05	0.05	0.94	1.01	0.03	0.03	0.94
α_{15}	0.5	0.52	0.08	0.08	0.92	0.51	0.05	0.05	0.93	0.51	0.04	0.04	0.94
α_{16}	1.0	1.03	0.07	0.07	0.92	1.01	0.05	0.05	0.93	1.00	0.03	0.03	0.95
α_{19}	0.5	0.51	0.07	0.07	0.93	0.51	0.05	0.05	0.95	0.50	0.03	0.03	0.94
α_{20}	1.0	1.03	0.07	0.07	0.92	1.01	0.05	0.05	0.93	1.01	0.03	0.03	0.94

SE, standard deviation of estimates over 1000 replications; SEE, average of estimated standard errors over 1000 replications; CP, the empirical coverage probability of a nominal 95% confidence interval.

Appendix I Real Data Analyses: Additional Results

This section contains additional results for the three real data analyses, where the standard errors and change in BIC are included for the BAMLSS, SPR-SIC and ALASSO-IC methods. (Note that BAMLSS does not produce standard errors).

- Table I32 is analogous to Table 7 of the main paper for the prostate cancer data.
- Table I33 is analogous to Table 8 of the main paper for the sniffer data.
- Table I34 is analogous to Table 9 of the main paper for the Boston house price data.

Table I32: Prostate Cancer Data: estimation metrics

	BAMLSS				SPR-SIC			ALASSO-IC		
	$\hat{\beta}_j$	ΔBIC	$\hat{\alpha}_j$	ΔBIC	$\hat{\beta}_j$	ΔBIC	$\hat{\alpha}_j$	$\hat{\beta}_j$	ΔBIC	$\hat{\alpha}_j$
`inter`	-0.79		2.66		-0.78(0.61)		-0.73(0.14)	-0.27(0.63)		-0.68(0.15)
`lcavol`	**0.52**	35.56	-0.19		**0.53**(0.07)	36.71		**0.54**(0.08)	36.71	
`lweight`	**0.81**	11.82	**-0.93**	-1.23	**0.66**(0.17)	9.21		**0.52**(0.18)	9.21	
`svi`	**0.73**	5.09	0.77		**0.67**(0.20)	5.64		**0.53**(0.21)	5.64	
`age`	-0.01		0.02							
`lbph`	0.06		0.05							
`lcp`	-0.16		0.45							
`gleason`	0.02		-0.12							
`pgg45`	0.01		-0.01							

Significant effects indicated in bold.

Table I33: Sniffer Data: estimation metrics

	BAMLSS				SPR-SIC			ALASSO-IC		
	$\hat{\beta}_j$	ΔBIC	$\hat{\alpha}_j$	ΔBIC	$\hat{\beta}_j$	ΔBIC	$\hat{\alpha}_j$	$\hat{\beta}_j$	ΔBIC	$\hat{\alpha}_j$
`inter`	-1.20		-0.96		0.45(1.01)		2.01(0.13)	0.21(1.04)		2.03(0.13)
`gaspres`	3.34		**-3.46**	-1.51	**10.84**(1.51)	38.43		**9.79**(1.63)	28.45	
`gastemp`	**0.26**	70.75	**0.09**	-4.81	**0.15**(0.04)	12.97		**0.19**(0.04)	15.37	
`tanktemp`	**-0.15**	8.45	0.01					-0.07(0.05)	-2.00	
`tankpres`	2.69		**2.72**	-3.24	**-5.73**(1.23)	15.33		**-4.08**(1.58)	1.71	

Significant effects indicated in bold.

Table I34: Boston House Price Data: estimation metrics

	BAMLSS				SPR-SIC			ALASSO-IC		
	$\hat{\beta}_j$	ΔBIC	$\hat{\alpha}_j$	ΔBIC	$\hat{\beta}_j$	ΔBIC	$\hat{\alpha}_j$	$\hat{\beta}_j$	ΔBIC	$\hat{\alpha}_j$
`inter`	10.51		-2.39		13.26(0.36)		-3.30(0.06)	13.18(0.36)		-3.28(0.06)
`rooms`	**0.26**	160.18	**-0.20**	-2.52	**0.10**(0.02)	29.60		**0.10**(0.02)	29.45	
`lowstat`	**-0.02**	94.40	**0.03**	-1.26	**-0.03**(0.00)	193.18		**-0.03**(0.00)	193.02	
`stratio`	**-0.02**	53.39	-0.01		**-0.04**(0.00)	55.96		**-0.04**(0.00)	55.81	
`lproptax`	**-0.16**	40.46	0.45		**-0.26**(0.05)	24.78		**-0.25**(0.05)	24.62	
`ldist`	**-0.11**	35.59	-1.21	25.06	**-0.28**(0.03)	62.36		**-0.27**(0.03)	62.20	
`crime`	**-0.01**	25.81	-0.01		**-0.01**(0.00)	76.71		**-0.01**(0.00)	76.55	
`lnox`	**-0.28**	18.01	-1.19		**-0.62**(0.09)	34.87		**-0.60**(0.10)	34.72	
`radial`	**0.00**	10.76	**0.05**	21.61	**0.01**(0.00)	24.54		**0.01**(0.00)	24.38	

Significant effects indicated in bold.
